# Rehabilitation Approaches for Proximal Peroneal Tendinopathy With Concurrent Anterior Cruciate Ligament (ACL) Sprain: A Case Report

**DOI:** 10.7759/cureus.69706

**Published:** 2024-09-19

**Authors:** Amisha P Zade, Swapnil U Ramteke, Ashish Keoliya, Tanushree V Deshmukh

**Affiliations:** 1 Musculoskeletal Physiotherapy, Ravi Nair Physiotherapy College, Datta Meghe Institute of Higher Education and Research, Wardha, IND; 2 Sports Physiotherapy, Ravi Nair Physiotherapy College, Datta Meghe Institute of Higher Education and Research, Wardha, IND

**Keywords:** functional rehabilitation, lower extremity functional scale (lefs), peroneal tendinopathy, physical therapy, tendinopathy treatment

## Abstract

Proximal peroneal tendinopathy is a relatively rare condition and can cause severe pain, discomfort, and often disability, especially if it coexists with other pathologies of the lower limbs. This case report discusses a 32-year-old male surgeon with the condition of chronic lateral ankle pain due to proximal peroneal tendinopathy, complicated by an anterior cruciate ligament sprain, a posterior cruciate ligament ganglion cyst, and early medial meniscus degeneration. His ability to perform surgeries has been compromised due to the need to stand for prolonged periods. A multi-factorial rehabilitation approach was designed to address the tendon pathology and the knee dysfunctions associated with it. The treatment program included patient education, therapeutic exercise, and pain management. There were considerable improvements in terms of pain, range of motion, muscle strength, and function from the previous stage of six weeks. This case indicates the clinical necessity of kinetic-chain-oriented, very individualized approaches to rehabilitation for complex musculoskeletal conditions such as this, especially in active professionals. The success of the rehabilitation lies in the potential for recovery even with some tough conditions if the treatment modality is appropriately designed to address the interactive aspects of the patient's condition.

This case highlights the importance of an integrated approach in managing complex peroneal tendonitis, especially when associated with upstream joint pathology. The positive outcome underscores the value of early recognition and individualized management to restore function and avoid permanent complications. Additionally, the report serves as a guide for future research into the potential long-term benefits of holistic, patient-specific approaches for similar cases.

## Introduction

The peroneal muscles, situated in the lateral compartment of the lower leg, are essential for maintaining ankle stability. These muscles are innervated by the superficial peroneal nerve and originate from the lateral aspects of the tibia and fibula. They run along the posterior aspect of the ankle before inserting on the medial cuneiform and the base of the first metatarsal [1,2].

Peroneal tendon pathology includes a spectrum of conditions such as tendinopathy, subluxation, dislocation, and tendon tears. Among these, peroneal tendinopathy is particularly prevalent, characterized by inflammation and degeneration of the peroneal tendons. It is a frequent cause of lateral ankle pain, especially in athletes and individuals who engage in repetitive lower extremity activities [3].

Despite its prevalence, peroneal tendinopathy is often underdiagnosed, as its symptoms can resemble those of other conditions like lateral ankle sprains. The etiology of peroneal tendinopathy is multifactorial, involving intrinsic factors such as anatomical variations and biomechanical misalignments, and extrinsic factors like improper footwear or excessive training loads [4].

If left untreated, chronic peroneal tendinopathy can lead to significant tendon degeneration, increasing the risk of tendon rupture and long-term functional impairment [5]. While conservative management, including physical therapy and activity modification, is usually effective, surgical intervention may be necessary in more severe cases.

It's important to note that *tendinopathy* is an umbrella term that covers various chronic tendon disorders, including tendinosis, tendinitis, and tenosynovitis [6,7]. Tendons are highly structured connective tissues, primarily composed of collagen fibers and proteoglycans within a well-organized extracellular matrix (ECM), which contribute to their tensile strength and viscoelastic properties [8,9].

Chronic tendinopathy is often linked to repetitive microtrauma, which can interfere with the normal healing process and lead to pathological changes within the tendon. These changes may include disorganized collagen fibers, scar tissue formation, and calcifications, all of which can impair the tendon's mechanical properties and hinder its ability to heal properly [10].

The focus of this case report is on investigating the assessment, treatment, and outcomes of a patient diagnosed with phase I fibularis tendinopathy. It demonstrates how crucial a rehab program designed to restore function and address the root cause is in ensuring the issue does not return.

## Case presentation

**Figure 1:**
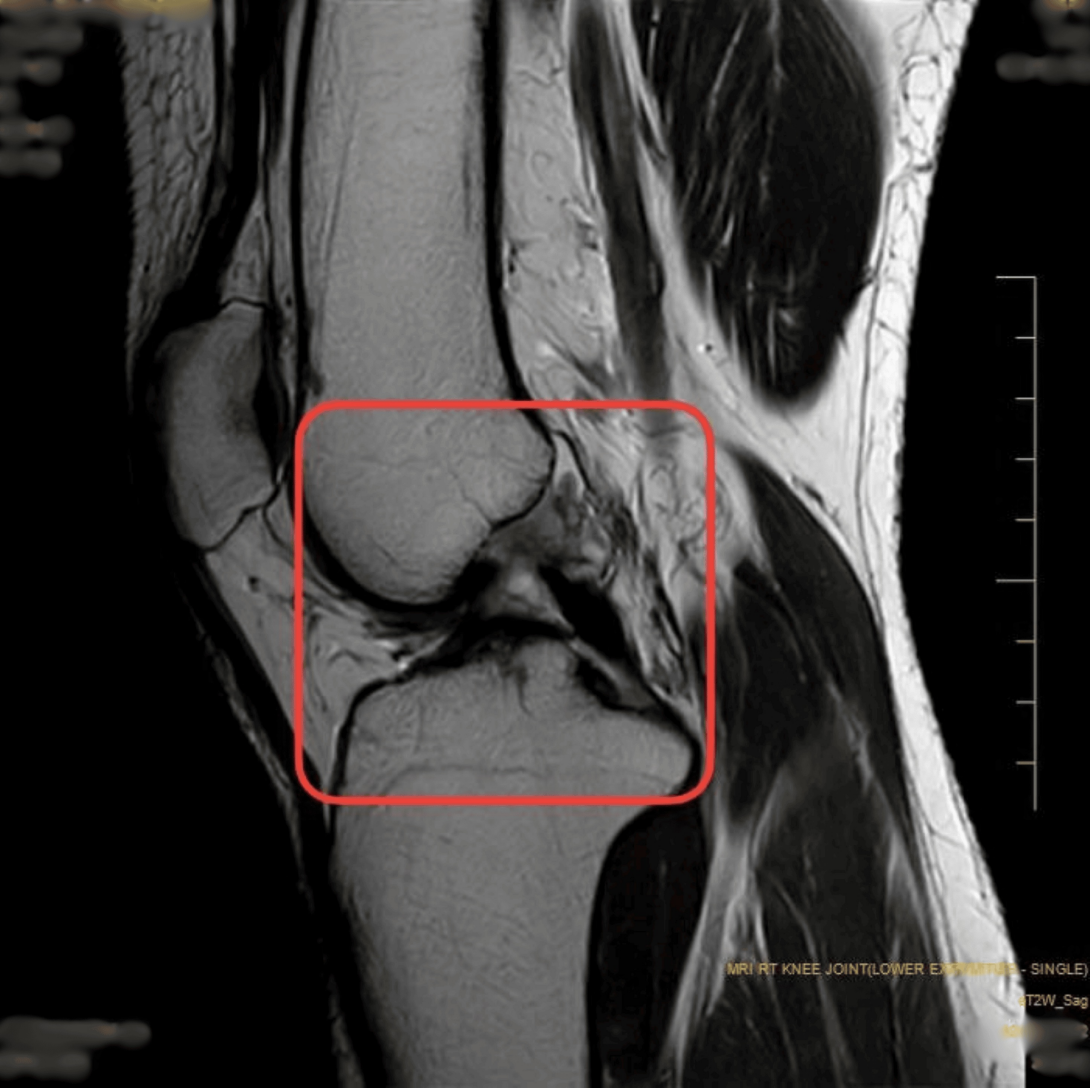
MRI of the right knee suggests a partial tear of the anterior cruciate ligament (ACL) with associated ligamentous strain.

**Figure 2:**
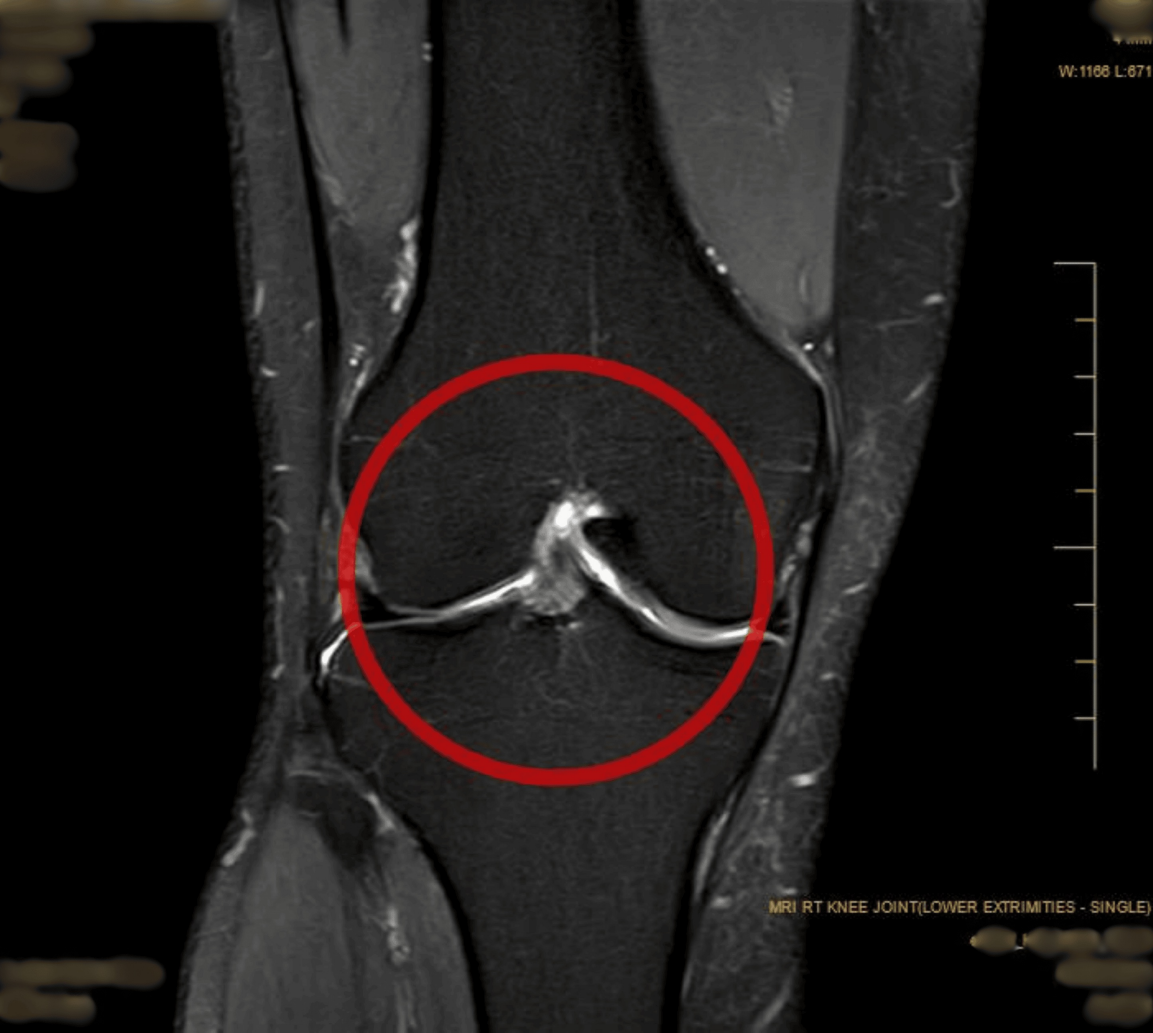
MRI of the right knee, coronal view, of a posterior cruciate ligament (PCL) ganglion cyst suggesting intrameniscal degeneration without a definitive tear.

A 32-year-old male surgeon was referred to the sports physical therapy department with complaints of persistent right lateral ankle pain that had been troubling him for the past six months. The pain began insidiously on January 4, 2024. He visited a private hospital in Pune and was advised to rest and take non-steroidal anti-inflammatory drugs (NSAIDs). However, his symptoms gradually progressed and were worsening, particularly during prolonged standing and sportive activities. The patient reported a persistent, dull, aching pain that had intensified over the past few weeks and was now present even at rest, including during sleep. He also noted some swelling around the lateral malleolus, accompanied by occasional clicking sounds. Despite no significant medical or surgical history, the patient has been recurrently using NSAIDs and applying ice with minimal relief. He visited Acharya Vinoba Bhave Rural Hospital (AVBRH) on June 11, 2024, where he underwent an MRI examination on June 13, 2024. The MRI revealed an anterior cruciate ligament (ACL) sprain, indicating ligamentous stress without a complete tear, a posterior cruciate ligament (PCL) ganglion cyst, and a grade 2a signal in the body and posterior horn of the medial meniscus, suggesting intra meniscal degeneration without a definitive tear. The same can be seen in Figures 1-2. These findings highlighted underlying knee pathology that could be contributing to or exacerbating the patient's peroneal tendinopathy. The patient was subsequently referred to the sports department, where pre-rehabilitation was initiated on August 26, 2024. The timeline of events is mentioned in Table 1.

**Table 1:** Patient's timeline of injury and treatment. AVBRH, Acharya Vinoba Bhave Rural Hospital

Events	Date
Visited a private hospital in Pune	January 04, 2024
Visited AVBRH	June 11, 2024
Investigatory findings were done	June 13, 2024
Initiation of pre-rehabilitation	July 26, 2024

Clinical findings

Before the physical examination, the patient provided both written and verbal informed consent. This included a detailed explanation of the examination procedures and the purpose of the assessment. During examination, the ankle had swelling or ecchymosis on the lateral aspect, and alignment was normal. Still, he had tenderness over his fibularis longus and brevis tendons, mild limitations in dorsiflexion, and weakness during foot eversion, consistent with the possibility of fibularis tendinopathy. Initial ultrasound revealed mild tendinopathy with no tears. There is a proposed management plan, and investigators are looking at activity modification, directed physical therapy focusing on conditioning the muscles supporting those affected, instruction about wearing appropriate shoes, and seating adjustments during work. Range of motion and manual muscle testing are mentioned in Tables 2-3.

**Table 2:** Ankle and knee range of motion pre- and post-rehabilitation.

Movement	Pre-rehabilitation	Post-rehabilitation
Ankle dorsiflexion	0-10°	0-15°
Ankle plantarflexion	0-40°	0-45°
Ankle inversion	0-25°	0-30°
Ankle eversion	0-10°	0-15°
Knee flexion	0-130°	0-135°
Knee extension	135°-0°	135°-0°

**Table 3:** Manual muscle testing of lower extremity.

Muscle	Movement	Pre-treatment	Post-treatment
Peroneal muscles	Foot eversion	3/5	4/5
Tibialis anterior	Ankle dorsiflexion	4/5	4/5
Gastrocnemius/Soleus	Ankle plantarflexion	4/5	5/5
Quadriceps	Knee extension	4/5	5/5
Hamstrings	Knee flexion	4/5	5/5

Diagnostic imaging

The MRI scan revealed a strain in the ACL, indicating it is stretched but not completely torn (Figures 1-2). Additionally, a fluid-filled cyst was found on the PCL. There was also evidence of early degeneration in the medial meniscus, specifically in the middle and back part, but no definitive tear was detected.

Therapeutic intervention

The physiotherapy intervention was designed using the International Classification of Functional Disability and Health (ICF) format. This format helped to focus on the patient’s specific physical limitations and how they affected their daily activities, as shown in Tables 4-8.

**Table 4:** Identified structural issues and corresponding interventions. ACL, anterior cruciate ligament

Codes	Structural impairment	Source of information	Clinical reason
s75021	Structure of ankle joint	MRI	- Manual therapy to address any soft tissue restrictions.
s75011	Structure of knee joint	MRI	- ACL rehabilitation protocol, including controlled strengthening and range-of-motion exercises.
s75010	Structure of lower leg muscles	On observation	- Targeted strengthening of the peroneal muscles and supporting musculature. - Soft tissue mobilization to reduce muscle tightness

**Table 5:** Functional limitations and their impact on daily activities. VAS, visual analog scale; ROM, range of motion; MMT, manual muscle testing; ACL, anterior cruciate ligament

Codes	Functional impairment	Source of information	Clinical reason
b28015	Pain in the lower limb	VAS scale, physical examination	The patient reports persistent dull aching pain, exacerbated by standing and physical activity, impacting daily function.
b7100	Reduced joint mobility	ROM	Limited ROM in ankle dorsiflexion and eversion, along with reduced knee flexion/extension, due to tendinopathy and ACL sprain.
b7300	Decreased muscle strength	MMT	Weakness in the fibularis muscles due to disuse and pain, leading to compromised functional performance.

**Table 6:** Impact of injury on patient's activities and role.

Codes	Participation restriction	Clinical reason
d850	Limited ability to perform job duties as a surgeon	The patient experiences difficulty standing for prolonged periods and performing precise movements due to ankle pain and knee instability, which are essential for surgical tasks. This limitation directly impacts his ability to fulfill his professional responsibilities.
d9201	Reduced participation in recreational activities	Due to persistent pain and the risk of exacerbating his condition, the patient has limited his engagement in recreational activities such as running, sports, or other physical hobbies. This restriction affects his quality of life and social interactions.
d901	Limited participation in community activities	The patient’s condition limits his ability to engage in community events or social gatherings that involve physical activity or prolonged standing. This restriction may contribute to social isolation and reduced community involvement.

**Table 7:** Activity limitation. ROM, range of motion; ACL, anterior cruciate ligament

Codes	Activity limitations	Source of information	Clinical reason
d4500	Difficulty walking short distances	Assessment	Pain and reduced ROM
d4552	Inability to run	Assessment	The patient avoids running and sports due to pain, fear of injury, and instability caused by the ACL sprain and tendinopathy.
d4453	Difficulty with climbing stairs	Assessment	Pain and reduced strength in the affected limb make climbing stairs challenging.

**Table 8:** Environmental and personal factors influencing recovery. NSAID, non-steroidal anti-inflammatory drug

Contextual factors	Codes	Clinical reason
Environmental factors	E110	NSAIDs are used for pain relief but with minimal effectiveness, indicating a need for additional pain management strategies.
	E410	Family support enhances recovery by providing emotional and practical assistance.
Personal factors	pf	Need psychological support
	pf	Need family support

The physiotherapy intervention for a patient with proximal peroneal tendinopathy begins with a thorough initial assessment focusing on pain levels, range of motion, strength, and functional abilities. Phasic intervention is shown in Table 9.

**Table 9:** Rehabilitation phases and interventions.

Phases	Goals	Interventions	Intensity
Phase 1 (Weeks 1-2)	-Reduce pain and inflammation -Improve joint mobility -Begin muscle activation and strength-building	-Hot pack: Promote relaxation and blood flow -Percussion therapy: Alleviate muscle tension and enhance mobility -Cryotherapy: Manage inflammation and pain post-exercise -Therapeutic exercises: Initiate calf raises, lunges, and resistance band eversion strengthening -Balance training: Begin on an unstable surface -Patient Education: Activity modifications and home exercise program	-Hot pack: 15-20 min -Percussion therapy: 5-10 min -Cryotherapy: 10-15 min post-exercise -Calf raises: 3 sets × 10-15 reps -Resistance band eversion: 3 sets × 10-15 reps -Balance training: 5-10 min -SLR in four plains -2-3 sessions/week with daily home exercises
Phase 2 (Weeks 3-4)	-Increase strength and proprioception -Enhance functional movement patterns	-Continue hot pack and percussion therapy: As needed based on symptom response -Cryotherapy: Post-exercise to control inflammation -Progressive therapeutic exercises: Increase resistance in calf raises, lunges, and eversion strengthening -Advanced balance training: Introduce more challenging tasks on unstable surfaces	-Continue as in Phase 1, with progression: -Increase resistance/weights -Balance training: 10-15 min -2-3 sessions/week with updated home exercises lunges (right leg internal rotation): 3 sets × 8-10 reps -Goblet squats -By the end of the phase gradual initiation of plyometric training
Phase 3 (Weeks 5-6)	-Maximize strength and functional ability -Prepare for return to full activity	-Reassessment: Evaluate progress in pain, ROM, strength, and function -Functional Exercises: Include sport/activity-specific drills -High-level balance and proprioception training: Complex tasks, multi-plane movements	-Functional exercises: 3 sets × 10-15 reps -Balance training: 15-20 mins -Continue hot pack, percussion, and cryotherapy as needed -2-3 sessions/week with long-term home exercise plan

Outcome measure

An evaluation was then performed following an intensive four-week therapeutic intervention program. Results Post-rehabilitation ROM and MMT of all joints are shown in Tables 2 and 3. Outcome measures were assessed before and after rehabilitation using the lower extremity functional scale, a visual analog scale (VAS), Foot and Ankle Outcome Score (FAOS), and the Biodex Balance System Y-Balance Test. Pre- and post-treatment assessment results indicated clinically meaningful changes across all evaluations. Improvements were observed in both the gait, with an increased FGA score suggesting improvement in gait stability and functional mobility and a marked reduction in VAS scores for pain. In addition, with the FAOS they have improved significantly in terms of foot and ankle functionality, which has an impact on their quality of life.

Y-Balance Test

The results indicated that the rehabilitation program achieved desirable outcomes concerning balance and proprioception, as evidenced by the enhanced lower limb stability shown in Table 10. The scores of outcome measures were found to be increased post-rehabilitation.

**Table 10:** Outcome measures used during rehabilitation. FGA, Functional Gait Assessment; VAS, Visual Analog Scale; LEFS, Lower Extremity Functional Scale; FAOS, Foot and Ankle Outcome Score

Outcome measure	Treatment score	Treatment score	Interpretation
FGA	14/30	25/30	Marked reduction in pain intensity, reflecting effective pain management and patient comfort.
VAS	7/10	1/10	Marked reduction in pain intensity, reflecting effective pain management and patient comfort.
LEFS	45/80	70/80	Marked improvement in lower extremity functional abilities and overall mobility.
Balance and Proprioception Balance Test	75% reach on the affected side	90% reach on the affected side	Enhanced balance and proprioception, demonstrating improved lower limb stability and control.
FAOS	60/100	80/100	A notable improvement in foot and ankle function, symptoms, and quality of life, indicating positive treatment outcomes.

## Discussion

Fibularis tendinopathy, particularly when it involves the proximal tendon, is a condition often associated with overuse and biomechanical imbalances. The patient’s occupation, involving prolonged standing, likely contributed to the condition, as suggested by previous studies that link repetitive strain and improper loading with tendinopathy [5].

The initial ultrasound findings, which indicated mild tendinopathy without tears, aligned with typical presentations described in the literature, where tendinopathy is characterized by tendon thickening and degeneration without complete rupture [11]. The MRI findings of knee pathology were particularly noteworthy. The ACL sprain and PCL ganglion cyst, coupled with medial meniscal degeneration, likely contributed to altered biomechanics, exacerbating the fibularis tendinopathy. This relationship between knee pathology and distal lower limb tendinopathies has been supported by studies emphasizing the impact of knee joint instability and alignment issues on ankle and foot mechanics [12].

Posttreatment assessments revealed marked improvements in both ROM and muscle strength, as demonstrated in the accompanying tables. These improvements were further demonstrated with functional outcome measures of the patient, including the Functional Gait Assessment, Pain VAS FAOS, and Balance and Proprioception Y-Balance Test. This decrease in pain (VAS) and increase in functional scores (FGA, LEFS, and FAOS) are consistent with other studies demonstrating the efficacy of conservative management, including physical therapy and ergonomic advice, in treating tendinopathy [13].

The course of the patient conveys the importance of a multidisciplinary way to deal with treating many-sided cases containing numerous concurrent pathologies in joints. The treatment plan addressed both the proximal and distal factors that contributed to her symptoms in tandem, which allowed for a reduction of pain, and improvement in restoration function, keeping her active lifestyle with an overall better quality of life. This philosophy is consistent with the current proposed model of management, as described in much contemporary literature supporting individualized and comprehensive treatment strategies for tendinopathy [14].

Research done by Powers et al. supported the idea that addressing dysfunction within the kinetic chain can lead to more effective and lasting. Another example is reducing the forces to the tendons by correcting a misaligned gait, such as overpronation through strengthening of muscles, in this case around the hip and knee, making the alignment better. Likewise, interventions to improve foot or ankle kinematics may have a downstream functional effect on the knee joint and decrease associated pain and disability [15].

The systematic review done by Chen et al. analyzed the potential effects of foot orthoses on patellofemoral pain syndrome (PFPS), which reflects the relationship between strike-down ally mechanics and knee behavior.

Conclusions of the review

The physical therapy interventions for PFPS, including knee taping and foot orthotics, were more effective in aligning lateral patellar tilt or mal tracking than quadriceps strengthening but only by a minimal clinically important change despite using structured exercise programs [16]. The article by Xie et al. examined the biomechanical link between proximal factors (hip mechanics) and patellofemoral knee pain, stating that there are compensatory changes in foot and knee kinematics due to dysfunction in the hip. The results support the kinetic chain concept and its relevant influence on lower limb pathologies [17].

Kumar et al. and Palmitier et al. emphasized the importance of tailored rehabilitation programs that incorporate both closed-chain kinetic (CKC) and open-chain kinetic (OKC) exercises for optimizing recovery [18]. CKC exercises, such as squats and lunges, are favored for their comprehensive engagement of lower extremity muscles. These kinetic chain exercises help reduce ACL strain by promoting axial load orientation and muscular co-contraction, which are essential for joint stability during rehabilitation [19]. This integrated approach aids in restoring dynamic control of the knee joint, as demonstrated by Dolan et al.'s findings [20].

## Conclusions

This case report underscores the critical importance of a comprehensive approach in managing proximal fibularis tendinopathy, particularly when it is associated with knee pathologies. The positive outcomes achieved through a multidisciplinary rehabilitation program highlight the effectiveness of addressing both distal and proximal factors contributing to the condition. Future research should focus on exploring the long-term benefits of this holistic approach and assessing its applicability across a broader patient population.
